# ^99m^Tc-HDP Labeling—A Non-Destructive Method for Real-Time Surveillance of the Osteogenic Differentiation Potential of hMSC during Ongoing Cell Cultures

**DOI:** 10.3390/ijms232415874

**Published:** 2022-12-14

**Authors:** Jakob Hofmann, Kai Borcherding, Karsten Thiel, Thomas Lingner, Ulrike Sommer, Uwe Haberkorn, Tim Niklas Bewersdorf, Gerhard Schmidmaier, Tobias Grossner

**Affiliations:** 1 Center for Orthopaedics and Trauma Surgery and Paraplegiology, Clinic for Trauma- and Reconstructive Surgery, University Hospital Heidelberg, 69120 Heidelberg, Germany; 2 Frauenhofer Institute for Manufacturing Technology and Advanced Materials IFAM, 28359 Bremen, Germany; 3 Genevention GmbH, Rudolf-Wissell-Str. 28A, 37079 Göttingen, Germany; 4 Department of Nuclear Medicine, University Hospital Heidelberg, 69120 Heidelberg, Germany; 5 Clinical Cooperation Unit Nuclear Medicine, German Cancer Research Center (DKFZ), 69120 Heidelberg, Germany; 6 Translational Lung Research Center Heidelberg (TLRC), German Center for Lung Research (DZL), 69120 Heidelberg, Germany

**Keywords:** ^99m^Tc-HDP, osteogenesis, mesenchymal stem cells, technetium labeling, cell culture

## Abstract

99-Metastabil Technetium (^99m^Tc) is a radiopharmaceutical widely used in skeletal scintigraphy. Recent publications show it can also be used to determine the osteogenic potential of human mesenchymal stem cells (hMSCs) by binding to hydroxyapatite formed during bone tissue engineering. This field lacks non-destructive methods to track live osteogenic differentiation of hMSCs. However, no data about the uptake kinetics of ^99m^Tc and its effect on osteogenesis of hMSCs have been published yet. We therefore evaluated the saturation time of ^99m^Tc by incubating hMSC cultures for different periods, and the saturation concentration by using different amounts of ^99m^Tc activity for incubation. The influence of ^99m^Tc on osteogenic potential of hMSCs was then evaluated by labeling a continuous hMSC culture three times over the course of 3 weeks, and comparing the findings to cultures labeled once. Our findings show that ^99m^Tc saturation time is less than 0.25 h, and saturation concentration is between 750 and 1000 MBq. Repeated exposure to *γ*-radiation emitted by ^99m^Tc had no negative effects on hMSC cultures. These new insights can be used to make this highly promising method broadly available to support researchers in the field of bone tissue engineering using this method to track and evaluate, in real-time, the osteogenic differentiation of hMSC, without any negative influence on the cell viability, or their osteogenic differentiation potential.

## 1. Introduction

99-Metastabil Technetium (^99m^Tc) is a *γ*-emitting radionuclide used for a wide range of applications in nuclear medicine [1,2]. ^99m^Tc can bind to organic molecules, thus making them detectable by radioactive imaging devices such as gamma cameras or dose calibrators [3]. One of the earliest and most important fields of ^99m^Tc imaging is visualizing bone structures, and metabolic processes. This is known as skeletal scintigraphy [4,5]. In 1971, Subramanian and McAfee described the first ^99m^Tc-radiopharmaceutical widely used for bone labeling, ^99m^Tc-polyphosphate [6]. Indications for the use of skeletal scintigraphy include the surveillance of neoplasia such as sarcomas, detecting insufficiency fractures, and diagnosing metabolic bone disease such as osteomalacia [7,8]. Meanwhile, technetium has been bound to a number of phosphate and phosphonate compounds, including methylene diphosphonate (MDP), ethylene diphosphonate (EHDP), and hydroxydiphosphonate (HDP) [3,9]. These phosphonate compounds bind to hydroxyapatite via adsorption, thus linking ^99m^Tc to the calcium complex [10]. Hydroxyapatite (Ca_5_[(OH)(PO_4_)_3_] or Ca_10_[(OH)_2_(PO_4_)_6_) is secreted by osteoblasts, forms an extracellular matrix, and can therefore be used to characterize osteogenesis [11,12,13].

In recent years, it has been found that ^99m^Tc can be used to quantify the osteogenic potential of cell cultures in vitro [14,15]. This is highly relevant in bone tissue engineering (BTE). Among other things, BTE assesses the ability of cell lines to form bone tissue under different conditions [16]. In particular, osteogenic differentiation of human mesenchymal stem cells (MSCs) has become a standard procedure in BTE [11,17]. Osteogenic potential is usually determined by quantifying the amount of hydroxyapatite secreted, since approximately 65% of bone tissue is made up of inorganic mineral, and hydroxyapatite accounts for up to 85% of this extracellular matrix [13,18]. This quantification requires an easily available and highly sensitive method to detect hydroxyapatite. The current gold standard for detecting hydroxyapatite, as defined by the Society of Cellular Therapy, is histological alizarin red staining [19]. While this method allows the quantification of hydroxyapatite, it is an elaborate process that has the disadvantage of permanently destroying the cell culture. Furthermore, it is not suitable for monitoring the differentiation towards the osteogenic lineage during the ongoing cell culture [20]. However, labeling with ^99m^Tc-polyphosphonates does not require the termination of the cell culture [21]. Therefore, it is theoretically possible to conduct further experiments with the same cell culture after labeling, and monitoring the osteogenic differentiation during the running cell culture would also, probably, be possible. This would constitute a huge scientific and economic benefit for researchers. However, some radioactive nuclides are known to have adverse effects on cell cultures [22], and it is not known whether that is the case for ^99m^Tc. This is vital to know as such harm would preclude the use of ^99m^Tc for monitoring purposes. Therefore, this study was performed to assess the effect of repeated labeling with ^99m^Tc on cell viability, and the ability of cell cultures to retain their differentiation potential to form hydroxyapatite.

This promising method can only be used for labeling living cell cultures if the procedure can be performed quickly, as the labeling method requires the temporary removal of cell culture media, and a prolonged exposure to room temperature [14,15,23]. The incubation time needed to bind the ^99m^Tc-compound to the hydroxyapatite is, therefore, a limiting factor. Unfortunately, this has not yet been determined under experimental conditions. Previous publications used incubation times of 2 h, but the minimal incubation time needed may be substantially lower as the binding process of the tracer probably happens within minutes. Furthermore, an absolute quantification of hydroxyapatite content in a cell culture requires the existing mineral to be completely saturated with ^99m^Tc-HDP. However, little is known about the saturation concentration of ^99m^Tc-HDP when used for assessing hydroxyapatite in vitro, whereas it is important to know how much activity is needed to fully saturate a certain amount of mineral. In previous experiments, semi-quantitative labeling was performed using 5 MBq by default, but the required amount of initial activity may be substantially lower or higher [14,15,23].

Therefore, in the second part of this paper we present another study, which assessed the minimal incubation time and required radioactive activity for full saturation for labeling osteogenic cultures of hMSCs with ^99m^Tc-HDP.

## 2. Results

### 2.1. Experiment 1: ^99m^Tc-HDP Saturation Time Evaluation

#### 2.1.1. Corrected for Natural Radioactive Decay

**Figure 1:**
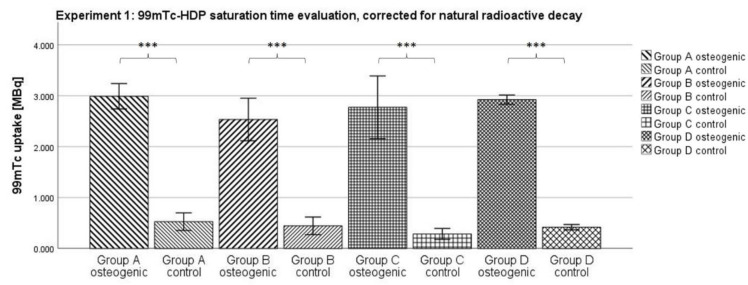
Mean ^99m^Tc-HDP uptake as measured by the dose calibrator when testing for the saturation time. The error bars show the standard deviation. Stars indicate significant differences between the two groups connected by a bracket. *** = significance *p* ≤ 0.001. *n* = 6.

Within the osteogenic groups, Group A showed the highest mean uptake with 2.988 MBq. Group B showed a mean uptake of 2.532 MBq, Group C of 2.771 MBq, and Group D of 2.923 MBq. Among the control groups, the highest mean uptake was 0.526 MBq in Group A. Group B showed a mean uptake of 0.444 MBq, Group C of 0.285 MBq, and Group D of 0.417 MBq (see Figure 1).

According to the Kolmogorov–Smirnov test, all groups showed a normal distribution. According to the ANOVA analysis, each osteogenic group showed a significantly higher uptake in ^99m^Tc-HDP compared to its corresponding control group. There was no significant difference between any of the osteogenic groups.

#### 2.1.2. Uncorrected for Natural Radioactive Decay

**Figure 2:**
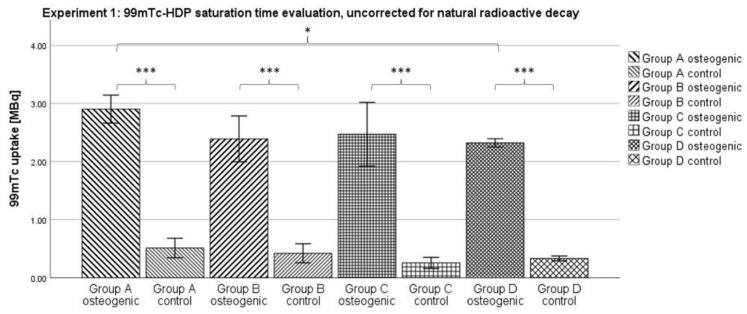
Mean ^99m^Tc-HDP uptake as measured by the dose calibrator when testing for the saturation time and not correcting for natural radioactive decay. The error bars show the standard deviation. Stars indicate significant differences between the two groups connected by a bracket. * = significance *p* ≤ 0.05; *** = significance *p* ≤ 0.001. *n* = 6.

When not corrected for the natural decay of ^99m^Tc, the uptake values showed a decrease from Group A to Group D. The mean uptake for the osteogenic groups was 2.903 MBq in Group A, 2.391 in Group B, 2.470 in Group C, and 2.32 in Group D. This resulted in a significantly lower uptake in Group D compared to Group A (see Figure 2).

The uncorrected mean uptake for the control groups was 0.511 in Group A, 0.419 in Group B, 0.254 in Group C, and 0.331 in Group D. All control groups still showed a significantly lower uptake than their corresponding osteogenic groups according to the ANOVA analysis. According to the Kolmogorov–Smirnov test, all groups showed a normal distribution.

### 2.2. Experiment 2: ^99m^Tc-HDP Saturation Concentration Evaluation

**Figure 3:**
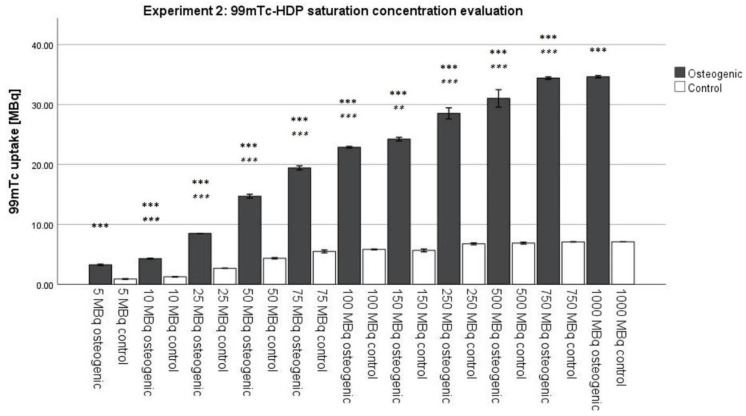
Mean ^99m^Tc-HDP uptake as measured by the dose calibrator when testing for the saturation concentration. The error bars show the standard deviation. Stars indicate significant differences: bold stars mean a significant difference between an osteogenic group and its corresponding control group. Stars in italics indicate a significant difference between an osteogenic group and the previous osteogenic group. ** = significance *p* ≤ 0.01, *** = significance *p* ≤ 0.001. *n* = 6.

For all osteogenic groups, an increase in initial activity was accompanied by an increase in ^99m^Tc uptake. There was, however, no linear correlation between initial activity and ^99m^Tc uptake. A 5-fold increase in initial activity from 10 MBq to 50 MBq resulted in only a 3.5-fold increase in ^99m^Tc uptake, from 4.290 MBq to 14.705 MBq. The increase in ^99m^Tc uptake slowed down even further for higher initial activities. The 5-fold increase from 100 MBq to 500 MBq in initial activity only resulted in a 1.4-fold increase in ^99m^Tc uptake, from 22.883 MBq to 31.033 MBq. The highest ^99m^Tc uptake of 34.649 MBq was finally reached with 1000 MBq of initial activity. However, this value was only different by 0.228 MBq from the uptake achieved by 750 MBq of initial activity. The uptake of the control groups also slowly increased with an increase in initial activity from 0.883 MBq (5 MBq initial activity) to 7.083 (1000 MBq initial activity) (see Figure 3).

According to the Kolmogorov–Smirnov test, all groups showed a normal distribution. According to the ANOVA analysis, each osteogenic group showed a significantly higher uptake in ^99m^Tc-HDP compared to its corresponding control group. All osteogenic groups were significantly different from each other except for the groups that received 750 MBq and 1000 MBq, respectively, in initial activity. They were not significantly different from each other.

### 2.3. Experiment 3: Evaluation of the Repeated ^99m^Tc-HDP Labeling on the Osteogenic Potential of hMSCs

#### 2.3.1. ^99m^Tc-HDP Labeling

**Figure 4:**
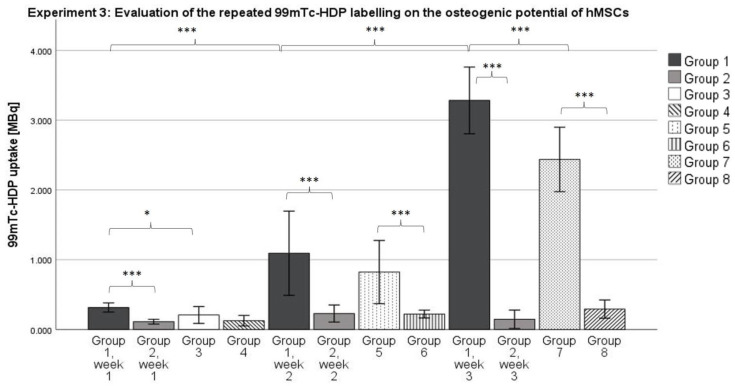
Mean ^99m^Tc-HDP uptake as measured by the dose calibrator. The error bars show the standard deviation. Stars indicate significant differences between the two groups connected by a bracket. * = significance *p* ≤ 0.05, *** = significance *p* ≤ 0.001. *n* = 6.

For Groups 1 and 2, there was an increase in ^99m^Tc uptake over the 3-week period. The labeling after 1 week showed a mean uptake of 0.315 MBq for Group 1, and of 0.111 MBq for Group 2. Groups 3 and 4, which were also labeled after 1 week, showed a mean uptake of 0.208 MBq and 0.126 MBq, respectively. According to the ANOVA analysis, Group 1 showed a significantly higher uptake at Week 1, compared to Group 3 (*p* ≤ 0.05) (see Figure 4).

The labeling after 2 weeks showed a mean uptake of 1.093 MBq for Group 1, and 0.228 MBq for Group 2. The corresponding Groups 5 and 6 showed mean uptakes of 0.822 MBq and 0.221 MBq, respectively. According to the ANOVA analysis, there was no difference between Groups 1 and 5 at Week 2. However, the uptake of Group 1 at Week 2 was significantly higher than the uptake of Group 1 at Week 1 (*p* ≤ 0.001) (see Figure 4).

The labeling after 3 weeks showed a mean uptake of 3.283 MBq for Group 1, and 0.146 MBq for Group 2. The corresponding Groups 7 and 8 showed mean uptakes of 2.437 MBq and 0.292 MBq, respectively. According to the ANOVA analysis, Group 1 showed a significantly higher uptake at Week 3 compared to Group 7 (*p* ≤ 0.001). The uptake of Group 1 at Week 3 was also significantly higher than the uptake of Group 1 at Week 2 (*p* ≤ 0.001) (see Figure 4).

According to the Kolmogorov–Smirnov test, all groups showed a normal distribution. According to the ANOVA analysis, each osteogenic group showed a significantly higher uptake of ^99m^Tc-HDP compared to its corresponding control group, with one exception: there was no significant difference between Groups 3 and 4 at Week 1.

#### 2.3.2. DAPI Cell Count

**Figure 5:**
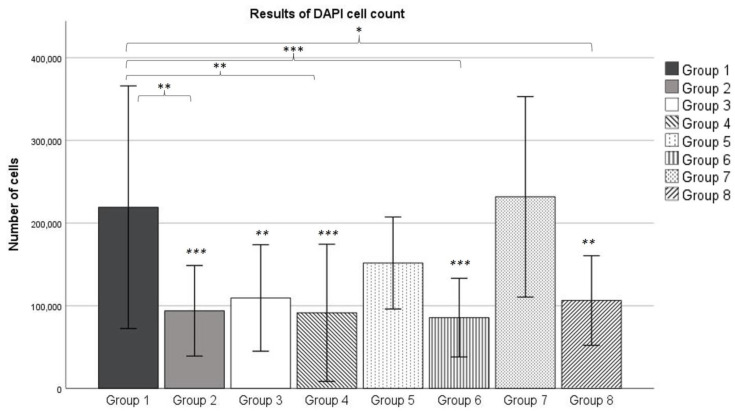
Mean number of cells counted after DAPI staining. The error bars show the standard deviation. Stars indicate significant differences: stars above brackets show a significant difference between the two groups linked, stars in italics show a significant difference with Group 7. * = significance *p* ≤ 0.05, ** = significance *p* ≤ 0.01 *** = significance *p* ≤ 0.001. *n* = 3.

The mean number of cells counted per group ranged from 85,513 (Group 6) to 231,694 (Group 7). Within the osteogenic Groups 3, 5 and 7, the number of cells increased over time from 109,349 in Week 1 (Group 3) to the aforementioned 231,694 in Group 7. Group 1 had a mean cell count of 219,103 in the third week. All osteogenic groups showed a higher number of cells than their corresponding control group (see Figure 5).

According to the Kolmogorov-Smirnov test, all groups showed a normal distribution. The ANOVA analysis revealed no significant difference between Groups 1 and 7. The higher number of cells in the osteogenic groups, compared to their corresponding control group, was significant for Groups 1 (compared to Group 2) and 7 (compared to Group 8). Additionally, Group 7 showed a significantly higher uptake than Group 3.

#### 2.3.3. Electron Microscopy

**Figure 6:**
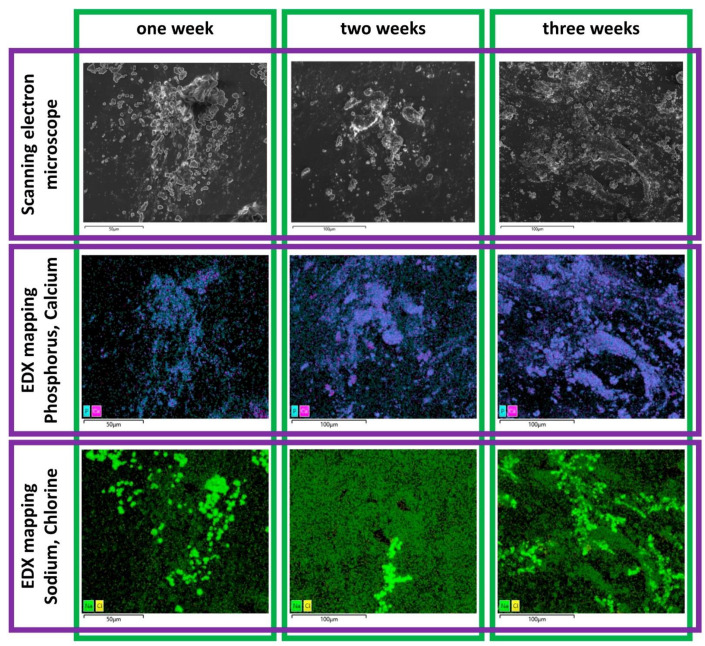
Scanning electron microscopy images of the mineralization study at the different time points and corresponding energy-dispersive X-ray spectroscopy mapping for Na, Cl and Ca, P.

On all SEM images, crystalline structures and dried cell structures were identified. On separate structures, the EDX mapping showed an allocation of the elements Ca, P, Na and Cl. The other elements identified were not assigned to specific regions of the structures (see Figure 6).

The elemental ratio of Ca/P for the time point “1 week” was 1.16, calculated for the complete image. The EDX analysis of the elemental Ca- and P- rich structure revealed an elemental ratio of Ca/P of 1.32 +/− 0.28 (*n* = 3). For time point “2 weeks”, the elemental ratio of Ca/P was calculated as 1.38, and for 3 weeks, 1.53.

## 3. Discussion

Labeling with ^99m^Tc-HDP is a highly sensitive method to determine hydroxyapatite formation in vitro. It has already been shown that ^99m^Tc-HDP labeling can reliably quantify osteogenesis when compared to the gold standard of alizarin red staining [14].

When assessing the osteogenic potential of stem cells, it has always been disputed whether the osteogenically differentiated cells actually form hydroxyapatite, and not a related calcium complex to which ^99m^Tc-HDP can also bind [24]. We performed an SEM/EDX test on our samples to address this issue, and establish whether ^99m^Tc-HDP labeling is capable of marking actual hydroxyapatite.

The elemental distribution revealed two categories of structures. The cubic, crystalline-shaped structure with the presence of the elements Na and Cl indicated crystallized sodium chloride. The structure consisting mainly of the elements Ca, P and O indicated the generation of a calcium phosphate. With regards to the setup of the mineralization study, this finding can be interpreted as the presence of hydroxyapatite. The evaluated elemental ratio in this area of Ca/P 1.16–1.53 was in a comparable range for human bone, which was reported to be 1.39–1.62 [25]. The determined increasing elemental ratio of Ca/P from Week 1 to Week 3 could be related to the different status of mineralization, as reported by Prati et al. [26]. Overall, these findings can be interpreted as successful osteogenesis.

### 3.1. Experiment 1: ^99m^Tc-HDP Saturation Time Evaluation

When testing for the saturation time, no significant difference between any two of the osteogenic groups could be shown. Naturally, the uptake declined with time; due to the relatively short half-life of ^99m^Tc of 6 h, a substantial amount of radionuclide was lost to natural decay over the longest incubation time of 2 h, compared to the shortest of 0.25 h. However, all osteogenic groups showed similar uptakes after correcting for this effect. It seems that after an incubation time of 0.25 h, any additional time does not result in the binding of any additional radionuclide to the hydroxyapatite. Instead, the reaction takes place within the first 0.25 h. These findings correlate well with a study by Yoichi Okamoto who found that the full adsorption of ^99m^Tc-HDP occurs within the first 0.5 h after exposure [27].

The incubation time might even be shorter than 0.25 h and further experiments are needed to determine that. In clinical settings, skeletal scintigraphy is performed by taking images at three distinct time points: immediately after injection, after 2–10 min, and then again after 2–4 h. However, this practice is due to the distribution of the tracer in vivo, and is unrelated to the actual binding of the tracer to hydroxyapatite [28].

Using an incubation time of just 0.25 h makes the method of ^99m^Tc-HDP labeling even more efficient, and allows living cell cultures to be labeled more safely since they only need to be taken out of culture for a short period of time.

### 3.2. Experiment 2: ^99m^Tc-HDP Saturation Concentration Evaluation

Similar to the incubation time of 2 h, until now, a standard initial activity of 5 MBq was routine for ^99m^Tc-labeling. Our results showed that this activity is far too low when trying to achieve full saturation of the cell culture. Each osteogenic group was significantly different from the one receiving less activity up to the point of 1000 MBq of initial activity, which showed a significantly different uptake compared to the group receiving 750 MBq. Therefore, full saturation is reached somewhere between 750 MBq and 1000 MBq initial activity. The exact amount needs to be determined in further experiments. For an absolute quantification of hydroxyapatite, much higher activities (around 1000 MBq) need to be used. This is an extremely high amount of activity; almost double the amount the European Association of Nuclear Medicine recommends for skeletal scintigraphy [29]. Relative quantifications are, however, still possible with lower activities since they only require that all samples receive the same amount of initial activity. For most scientific questions, it is sufficient to establish the relative relationship between two or more groups. Our findings are limited by the fact that the saturation concentration strongly depends on the size of the cell culture. A high number of cells used for the experiment or osteogenically very potent cells may result in high amounts of hydroxyapatite formed, and thus require higher amounts of ^99m^Tc-HDP to achieve saturation [30]. Whether there is a generalizable correlation between cell culture size, osteogenic potential of the cells, and saturation concentration should be investigated in further experiments.

### 3.3. Experiment 3: Evaluation of the Repeated ^99m^Tc-HDP Labeling on the Osteogenic Potential of hMSCs

Considering the data we revealed, it can be stated that a significant difference between the osteogenic group and the control group can already be measured after 1 to 2 weeks (see differences between Groups 1 and 2 after 1 week, and 5 and 6 after 2 weeks) using the ^99m^Tc Labeling method. This is highly beneficial for further experiments because it means that a shorter runtime of the experiments of only 1 to 2 weeks would be required to proof the differentiation toward the osteogenic lineage instead of 3 weeks.

Group 1 showed a higher mean uptake compared to Group 2 for all time points, although this difference was only statistically significant in Week 1 (*p* ≤ 0.05) and Week 3 (*p* ≤ 0.001). It seems that the repeated labeling with ^99m^Tc-HDP does not result in a diminished osteogenic potential of the cell culture. Rather, Group 1 showed a higher osteogenic potential. This is particularly striking since all groups used cells from the same donors, so inter-donor differences cannot explain differences in osteogenic potential.

After 3 weeks of incubation, the DAPI cell count revealed no significant difference between the number of cells in Group 1 and Group 7. This reveals that the exposure to *γ* radiation emitted by technetium does not result in a diminished number of living cells. These results match findings which showed that low-dose ionizing radiation has no significant effect on MSC viability up to doses of 50 mGy [31].

Over the course of 3 weeks, the number of cells increased each week for the osteogenic Groups 3, 5 and 7. This increase was large enough for Group 7 to show a significantly higher number of cells, compared to Group 3. At the same time, the number of cells within the control groups remained relatively stable over the observed period of time. However, each osteogenic group showed a higher number of cells counted compared to its corresponding control group, although this effect was only statistically significant for Group 1 (compared to Group 2) and Group 7 (compared to Group 8) according to the ANOVA analysis. Both this effect and the continuously increasing number of cells in the osteogenic groups could be explained by the influence of dexamethasone. Dexamethasone is known to have an effect on the proliferation of MSCs. Wang et al. found that small doses of dexamethasone promote proliferation in MSCs isolated from umbilical cords [32]. This effect occurred for doses in the order of 10–9 mol/L, which is exactly the concentration we used for osteogenic differentiation (100 nM, see Section 4.4). Therefore, it can be assumed that dexamethasone also promoted differentiation in MSCs isolated from bone marrow.

The limited exposure to *γ* radiation does not seem to adversely affect the osteogenic potential of MSCs. The relatively short half-life of ^99m^Tc might play a role in this since the amount of activity is quite quickly reduced to background radiation levels. On the other hand, limited doses of *γ* radiation are known to stimulate the proliferation of mesenchymal stem cells from mouse brains, up to a dose of 100 mGy [33]. However, at the end of the 3-week differentiation period, the cell count revealed no significant difference between Groups 1 and 7. Therefore, the higher osteogenic potential of Group 1 cannot be explained by a higher number at the end of the differentiation period. However, it might be the case that the *γ*-radiation accelerated the proliferation in the early stages of differentiation, which would mean more cells earlier on, resulting in a higher osteogenic potential. Still, repeated labeling also means that cell cultures need to survive out of culture conditions for a prolonged time. This is because ^99m^Tc-HDP labeling requires the removal of cell culture media for washing purposes, and is often performed at room temperature since incubators suitable for radioactive samples are not always available in close proximity to ^99m^Tc generators. The fact that MSCs can exist out of culture conditions is supported by other studies which have found that MSCs can survive at room temperature, and without culture media for a limited amount of time [34].

### 3.4. Conclusion Summary

In conclusion, our data strongly confirm ^99m^Tc-HDP labeling as a simple, reliable and sensitive method to assess osteogenesis in vitro. We defined a new standard for the incubation time, as we found that 0.25 h or less is sufficient for the tracer to bind to hydroxyapatite. The saturation of the cell culture for an absolute quantification of hydroxyapatite requires very high initial activities of around 1000 MBq, but semi-quantitative measurements using lower activities are usually sufficient for daily laboratory routines. What effect these high activities have on the cell culture is not yet clear, but we found that repeated labeling with 5 MBq at room temperature does not adversely affect the osteogenic potential, or cell number of MSC cell cultures. Consequently, ^99m^Tc-HDP labeling can be used for “milestone evaluations”, i.e., the assessment of osteogenesis during an ongoing cell culture. With that, ^99m^Tc-HDP would be a very valuable method for BTE as it would enable the development of innovative osteo-regenerative therapies.

## 4. Materials and Methods

### 4.1. Study Design at a Glance

Bone marrow aspirate from *n* = 6 healthy donors was obtained from the proximal femoral cavity. The mononuclear cell fraction was then isolated by a Ficoll gradient. Next, monoclonal cells were expanded in T-150 polystyrene tissue culture flasks using DMEM-HG until 90% confluence was achieved. Cells were then subsequently expanded until a sufficient number of cells was reached.

For each donor, cells were then seeded with a density of 10,000 cells/cm^2^ in 38 35-mm flat-bottom Petri dishes, and received DMEM-LG as media. Cells in half of these dishes were differentiated osteogenically using the osteogenic supplements L-ascorbic acid, dexamethasone, and *β*-glycrol phosphate. The other half served as control and received only DMEM-LG.

**Figure 7:**
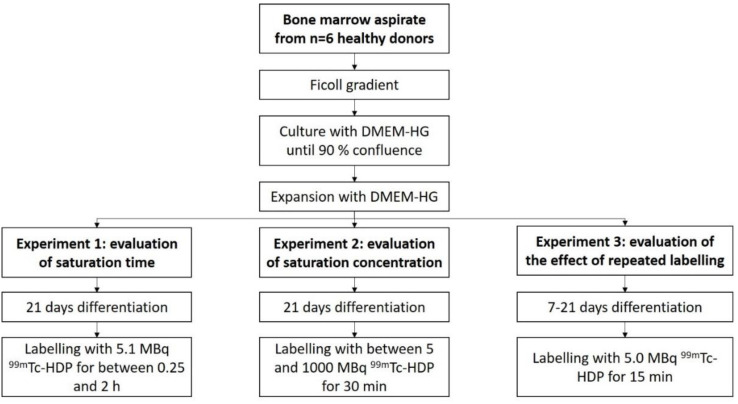
Overview of study design.

The dishes of each donor were then split for the three experiments: 1. ^99m^Tc-HDP saturation time evaluation (8 dishes), 2. ^99m^Tc-HDP saturation concentration evaluation (22 dishes) and 3. evaluation of the effect of repeated ^99m^Tc-HDP labeling on the osteogenic potential of hMSCs (8 dishes) (see Figure 7).

Experiment 1:

To evaluate the saturation time, the cells were differentiated for 3 weeks. They were then labeled with ^99m^Tc-HDP. For that, each dish received 5.1 MBq of activity in 1 mL 0.9% NaCl, and was incubated at room temperature. Group A was incubated for 0.25 h, Group B for 0.5 h, Group C for 1 h, and Group D for 2 h. After incubation, the dishes were washed with PBS, and bound activity was measured.

Experiment 2:

To evaluate the saturation concentration, the cells were differentiated for 3 weeks, and then labeled for 30 min at room temperature with different amounts of activity: 5 MBq, 10 MBq, 25 MBq, 50 MBq, 75 MBq, 100 MBq, 150 MBq, 250 MBq, 500 MBq, 750 MBq, and 1000 MBq. Bound activity was measured after incubation.

Experiment 3:

To evaluate the effect of repeated labeling, Groups 1 and 2 were differentiated for 3 weeks, and labeled after 1, 2, and 3 weeks (5.0 MBq, 15 min). Groups 3 and 4 were differentiated for 1 week, after which they were labeled (5.0 MBq, 15 min). Groups 5 and 6 were differentiated for 2 weeks, and labeled after that (5.0 MBq, 15 min), and Groups 7 and 8 were differentiated for 3 weeks, and then labeled (5.0 MBq, 15 min).

### 4.2. Harvest of Human Mesenchymal Stem Cells

Bone marrow aspirate from *n* = 6 healthy donors was obtained from the proximal femoral cavity under general anesthesia during an elective surgical procedure for total hip arthroplasty after informed consent was given. During the preparation of the proximal bone cavity, 10 mL of bone marrow was collected into a 20 mL syringe (BD, Heidelberg, Germany) containing 1000 IU of heparin (Braun, Melsungen, Germany). Each sample was then diluted 1:1 with PBS (Gibco, Frankfurt, Germany), and washed twice with PBS. The mononuclear cell fraction was isolated using a Ficoll gradient centrifugation (Ficoll-Paque-PLUS, Cytiva, Freiburg, Germany). These mononuclear cells were then plated in T-150 polystyrene tissue culture flasks (Falcon, Kaiserslautern, Germany) at a density of 5·10^5^ cells/cm^2^ and cultured in a humidified 5% CO_2_ atmosphere at 37 °C. The cell culture medium used was high-glucose Dulbecco’s modified Eagle’s medium (DMEM-HG, Gibco) containing 10% heat-inactivated (56 °C, 30 min) fetal bovine serum (FCS, Sigma, Schnelldorf, Germany), and 1% penicillin/streptomycin (Sigma). After 48 h, all samples were washed with PBS to remove nonadherent cells. All remaining adherent cells were defined as human bone marrow mesenchymal stem cells. Media were changed three times a week (every 2–3 days). At 90% confluence, cells were trypsinised (Trypsin-EDTA, Sigma) and stored as P0 cells for further experiments in liquid nitrogen. Each 0.5 mL aliquot contained 5·10^5^ cells in 10% DMSO (Sigma) at the time of freezing.

### 4.3. hMSC Expansion

P0 human MSCs (*n* = 6) were thawed and seeded into T-150 flasks (Falcon) with 500,000 cells each, and cultured for 10 days in a humidified 5% CO_2_ atmosphere at 37 °C. Media were changed every 2 days. The media used for expansion were DMEM-HG with 10% FCS and 1% penicillin/streptomycin. After 7 days, 90% confluence was reached in all cells cultured and the cells were trypsinised.

### 4.4. hMSC Differentiation

Cells from every donor (*n* = 6) were seeded and duplicated into 35-mm flat-bottom Petri dishes (Corning, Kaiserslautern) at a density of 10,000 cells/cm^2^. In total, 38 dishes were seeded per donor: 8 for the ^99m^Tc-HDP saturation time evaluation, 22 for the ^99m^Tc-HDP saturation concentration evaluation, and 8 for the evaluation of the effect of repeated labeling.

**Figure 8:**
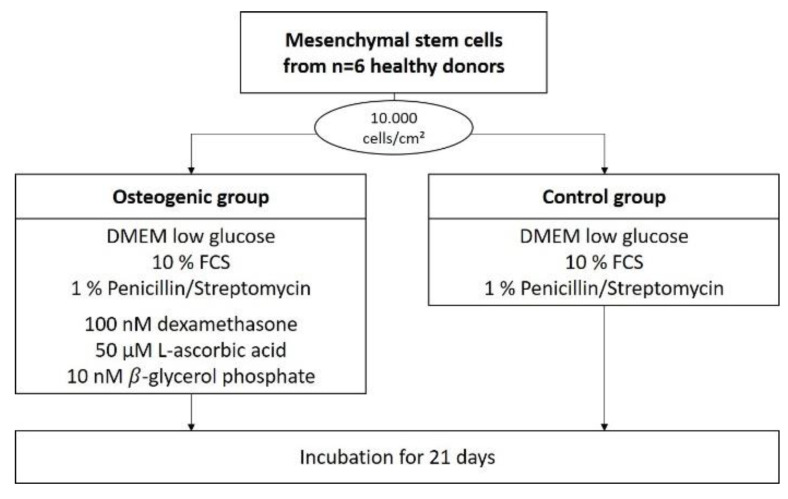
Flow chart for the differentiation of hMSCs.

Half of these dishes were treated with osteogenic medium, which was made up of DMEM low glucose containing 10% FCS, 1% penicillin/streptomycin and osteogenic supplements (100 nM dexamethasone, 50 µM ascorbic acid, 10 nM *β*-glycerol phosphate). The other half of the dishes were treated as a negative control and received media without osteogenic supplements (DMEM low glucose containing only 10% FCS and 1% penicillin/streptomycin) (see Figure 8).

If not otherwise specified, cells were cultured for 21 days in a humidified 5% CO_2_ atmosphere at 37 °C with media changed three times a week (every 2–3 days) and then terminated by being washed twice with PBS followed by air drying under a cell culture hood.

### 4.5. Experiment 1: ^99m^Tc-HDP Saturation Time Evaluation

**Figure 9:**
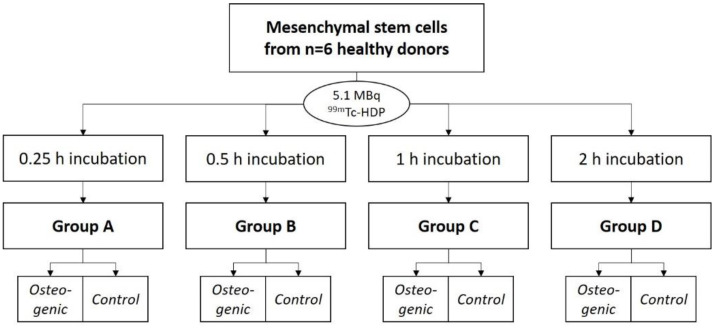
Study design for the ^99m^Tc-HDP saturation time evaluation.

For this experiment, 4 groups (*n* = 6) were formed. For each group and donor, two dishes were used: One with osteogenically differentiated cells and one with undifferentiated cells. A tracer aliquot of 5.1 MBq ^99m^Tc-HDP in 1 mL of 0.9% NaCl was added to each dish. Traced activity was determined with a dose calibrator (Activimeter ISOMED 1010, Nuklear-Medizintechnik Dresden GmbH, Dresden, Germany). Here, the dishes were directly placed into the detection chamber of the dose calibrator and the detection widow was set to Gamma decay/Technetium. The detection time was 5 s for each specimen. The dishes were then incubated at room temperature for different periods. One dish with differentiated and one dish with undifferentiated cells per donor were incubated for different amounts of time: Group A for 0.25 h, Group B for 0.5 h, Group C for 1 h and Group D for 2 h. After incubation, the remaining liquid ^99m^Tc-HDP was then removed, and the dishes were washed twice with PBS to remove any unbound tracer. Subsequently, the dishes were analyzed with the dose calibrator to precisely determine the amount of bound tracer (see Figure 9).

### 4.6. Experiment 2: ^99m^Tc-HDP Saturation Concentration Evaluation

**Figure 10:**
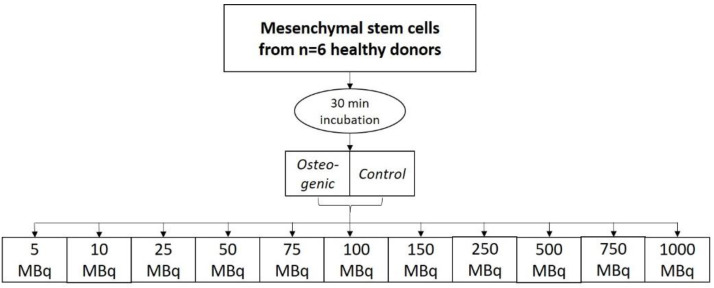
Study design for the ^99m^Tc-HDP saturation concentration evaluation.

For this experiment, 11 groups were formed (*n* = 6), with each group being again divided into an osteogenic and a control subgroup. Each group received different amounts of ^99m^Tc-HDP activity, which was determined in advance with a dose calibrator (Activimeter ISOMED 1010): 5 MBq, 10 MBq, 25 MBq, 50 MBq, 75 MBq, 100 MBq, 150 MBq, 250 MBq, 500 MBq, 750 MBq, 1000 MBq. The activity was dissolved in 1.0 mL 0.9% NaCl and then added to each respective dish. All groups were incubated at room temperature for 30 min. The remaining liquid ^99m^Tc-HDP was then removed, and the dishes were washed twice with PBS to remove any unbound tracer. After that, the dishes were analyzed with the dose calibrator to precisely determine the amount of bound tracer (see Figure 10).

### 4.7. Experiment 3: Evaluation of the Repeated ^99m^Tc-HDP Labeling on the Osteogenic Potential of hMSCs

For this experiment, for each of the *n* = 6 donors, 8 groups were formed. Group 1 received DMEM-LG with osteogenic supplements as media. The dishes of each donor were cultured in a humidified 5% CO_2_ atmosphere at 37 °C for 1 week. Then, they received a tracer aliquot of 5.0 MBq ^99m^Tc-HDP in 1 mL of 0.9% NaCl, and were incubated at room temperature for 15 min. The remaining liquid ^99m^Tc-HDP was then removed, and the dishes were washed twice with PBS to remove any unbound tracer. The dishes were then analyzed with the dose calibrator to precisely determine the amount of bound tracer. Here, the dishes were directly placed into the detection chamber of the dose calibrator (Activimeter ISOMED 1010, Nuklear-Medizintechnik Dresden GmbH, Dresden, Germany) and the detection widow was set to Gamma decay/Technetium. The detection time was 5 s for each specimen. After that, fresh media were added, and the dishes were cultured for another week. Subsequently, the dishes were labeled again (5.0 MBq, 15 min), and then for a third and final time after being cultured for a third week (5.0 MBq, 15 min).

Group 2 served as a control group to Group 1 and received DMEM-LG without osteogenic supplements as media, but was otherwise treated as Group 1.

**Figure 11:**
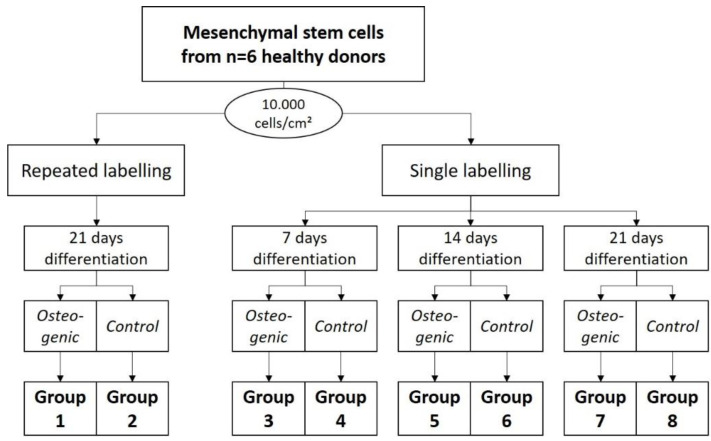
Study design for the evaluation of the repeated 99mTc-HDP labeling on the osteogenic potential of hMSCs.

**Figure 12:**
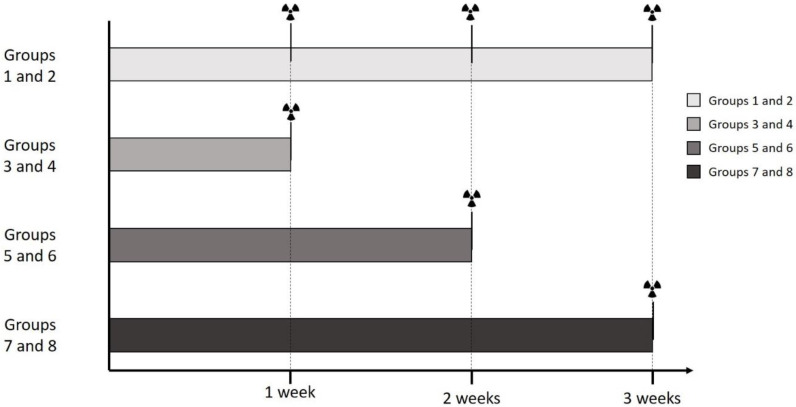
Schematic Overview for Groups 1 to 8 for a better overview of the individual timepoints when the radioactive labeling was performed. ☢ = Timepoint of radioactive labeling.

Groups 3–8 were labeled just once after being cultured for different time spans. The duration of culture was 1 week for Groups 3 and 4, 2 weeks for Groups 5 and 6, and 3 weeks for Groups 7 and 8. Groups 4, 6, and 8 served as control groups to the osteogenic Groups 3, 5, and 7. At the end of their respective time span, the culture was terminated, and the dishes were labeled with ^99m^Tc-HDP (5.0 MBq, 15 min). For the times of labeling, see also Figure 11 and Figure 12.

### 4.8. DAPI Staining and Cell Count

After the cell culture was terminated, dishes of repeated ^99m^Tc-HDP labeling (see Section 4.7) were incubated with 4% paraformaldehyde (PFA, Sigma) at room temperature for 20 min. They were then fixated with 0.1% PFA. All fixated samples were stained with DAPI (Thermo Fisher Scientific, Karlsruhe, Germany), and counted under a microscope (Leica CMi8, Leica, Wetzlar, Germany). Cells were counted at 100× magnification in 5 vision fields in 5 different areas of each dish (top left, top right, bottom left, bottom right, and center). Based on the specifications of the microscope, each vision field had an area of 952.484 µm^2^. As the area of an entire 35 mm dish was known, the absolute number of cells in the dish was calculated and assessed for normal distribution.

Cell counting in fluorescence images was conducted via the “CellProfiler” software (Broad Institute, “URL https://cellprofiler.org, version 4.2.4 accessed on 15 August 2022). Raw TIFF images were masked for time stamp and size legend, and converted to greyscale images. Cells were identified as primary objects using a global, manually set intensity threshold (t = 0.1), and objects smaller than 8 or larger than 80 pixels in diameter were disregarded. Clumped cells were distinguished based on the shape method.

### 4.9. Electron Microscopy

Scanning electron microscopy was carried out on dry samples from the mineralization study on a Helios Nanolab 600 machine (Thermo Fisher Scientific, Eindhoven, the Netherlands) with a resolution of about 1 nm. All images were acquired at an acceleration voltage of 5 kV with an Everhardt–Thornley secondary-electron detector. To avoid charging artefacts, 10 nm of carbon was deposited onto the sample surface prior to imaging. Elemental analysis with a focus on C, N, O, Na, Mg, P, S, Cl, K, and Ca was investigated by means of energy-dispersive X-ray analysis with an Oxford X-Max80 silicon-drift detector (Oxford Instruments, Abingdon, UK) attached to the SEM with an energy resolution of 124 eV at Mn-Kα.

### 4.10. Statistics

The results were first tested for normal distribution using the Kolmogorov–Smirnov test. To determine statistical significance between the groups, an ANOVA was performed followed by the adjustment of the p-value using the post hoc test by Bonferroni. The homogeneity of variances for one-way ANOVA was analyzed using Levene’s test to assess the equality of variances.

Statistical analyses were performed using SPSS Statistics^®^ (IBM, Armonk, NY, USA) Version 28. Statistical significance was set to *p* ≤ 0.05.

For the determination of the ^99m^Tc-HDP saturation time, measurements were corrected for the different incubation times regarding the decay of ^99m^Tc. For the formula, a half-life time of 6.0 h was used [35].

## Data Availability

There is no supplementary data available.
